# Mesothelioma in an HIV/AIDS patient without history of asbestos exposure: possible role for immunosuppression in mesothelioma: a case report

**DOI:** 10.1186/1757-1626-2-7498

**Published:** 2009-05-11

**Authors:** Cleve Orian James, Ashanti W Woods, Payam Arya, Khadega Ahmed Abuelgasim, Lekidelu Taddesse Heath, Amy Sitapati

**Affiliations:** 1 Department of Internal Medicine, Johns Hopkins University/Sinai Hospital2401 W. Belvedere Ave., Baltimore, MD 21215USA; 2 Department of Pediatrics, University of Maryland Medical Center22 South Greene St., Baltimore, MD 21201USA; 3 Department of Pathology, Howard University Hospital2041 Georgia Ave., Washington, DC 20060USA; 4 Department of Internal Medicine, Howard University Hospital2041 Georgia Ave., Washington, DC 20060USA; 5 Department of Pathology, Howard University Hospital2041 Georgia Ave., Washington, DC 20060USA; 6 Department of Internal Medicine, Howard University Hospital2041 Georgia Ave., Washington, DC 20060USA

## Abstract

We describe a 41-year-old African-American male who initially presented in respiratory distress. He had a positive history of asthma, cigarette smoking, and only recent possible asbestos exposure six months prior to onset of symptoms. Mesothelioma was suspected after chest radiography and PET-CT, and confirmed by immunohistochemical tissue analysis. We postulate that immunosuppression enhances susceptibility to mesothelioma, since weakened immune systems are present in both HIV/AIDS patients like this 41-year-old man, and elderly patients who compose the population that classically presents with mesothelioma. Furthermore, immunosuppression may be a prerequisite to the development of mesothelioma.

## Introduction

Mesothelioma is a type of cancer that affects the serous lining of the thorax, abdomen, or pelvis. Its incidence is 1 per 100 000 persons in the United States, and the 5-year survival is 3%. Mesothelioma classically occurs after the 5^th^ decade of life in individuals with a history of significant asbestos exposure that predates the cancer by 30-50 years. Disease risk is proportional to duration and dose of asbestos exposure. Initial clinical symptoms include shortness of breath and pleuritic chest pain, and imaging studies may reveal pleural/peritoneal effusions. The lifetime risk of developing mesothelioma after asbestos exposure is approximately 10%, however roughly 30% of mesothelioma victims do not have a history of asbestos exposure [1]. There does not appear to be a direct association between cigarette smoking and mesothelioma. This report describes advanced mesothelioma in a man who was relatively young and without significant asbestos exposure.

## Case presentation

The 41-year-old African-American man presented to the Emergency Department with severe shortness of breath, as well as severe, sharp, intermittent right-sided chest pain for 2-days duration without radiation. He had a history of asthma and HIV with progression to AIDS in the past 3 years. He also had a history of chronic alcohol and illicit drug use, and 20-pack years of cigarette smoking. Over the past 30-years, he worked in housekeeping at various hotels and occasionally as a shoe shiner. He did not have any occupational history of mining/milling, construction, ship-yard work or plumbing. The patient did have a brief history of assisting to demolish a house over an 8-hour period approximately 1 year previously; however it was unclear whether there was asbestos exposure during that time.

Physical examination revealed an anxious man in severe respiratory distress. His blood pressure was 157/86 mmHg; pulse rate 120/min; respiratory rate 40/min; temperature 97°F and oxygen saturation 92% on room air. He had reduced breath sounds throughout the left lung fields, with crackles in the right lower lung fields. The trachea was midline. He was tachycardic with normal heart sounds and a soft systolic murmur at the left lower sternal border. His abdomen was soft and without distention, tenderness or organomegaly. Capillary refill was normal, and Homan’s sign was negative. There was clubbing of his fingers. Neurologic examination was normal.

After sedating and intubating the patient, empiric antibiotics were initiated, and 1.6 L of serosanguinous pleuritic fluid was aspirated. Arterial blood gases on 100% oxygen via facemask revealed a blood pH of 7.42; PaCO_2_ 30 mmHg; PaO_2_ 80 mmHg; HCO_3_ 20 mEq/L and SaO_2_ 97%. Urine toxicology was positive for cocaine and opiates, while serum electrolytes, blood counts and liver function tests were normal. Cardiac enzymes were unremarkable, and electrocardiography showed sinus tachycardia with left ventricular hypertrophy and left atrial enlargement.

**Figure 1.**
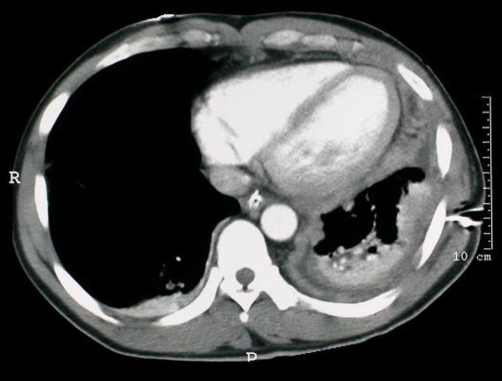
CT scan of the chest shows extensive left basilar pleural disease with loss of lung volume, nodular left pleural thickening and a nodular pericardium. Nodules in the right lung are consistent with metastatic disease.

**Figure 2.**
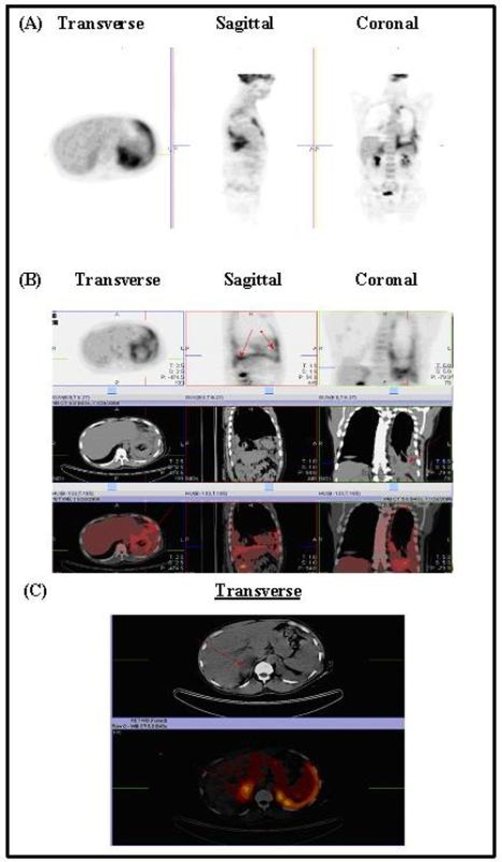
PET-CT shows extensive pleural thickening associated with hypermetabolism primarily in the lung base posteriorly, with a density that suggests excess pleural fluid **(A & B)**. There is a large mass in the right adrenal gland that is hypermetabolic and appears to be a tumor **(C)**.

Radiograph*y* of the chest revealed left basilar infiltrates and pleural density that could represent pleural effusion. Also, there were chronic right upper lung scarring and cystic changes. Computed Tomography of the chest and abdomen showed extensive left basilar pleural disease with loss of lung volume, nodular left pleural thickening and a nodular pericardium. Nodules in the right lung were consistent with metastatic disease (see Figure 1). The right adrenal gland contained a necrotic mass (not shown). Positron Emission Tomography-Computed Tomography (PET-CT) revealed extensive left pleural thickening with associated hypermetabolism. A greater degree of hypermetabolism was located in the posterior lung base, with a density that was interpreted as pleural fluid (see Figure 2A & B). The right adrenal mass demonstrated hypermetabolic activity (see Figure 2C).

**Figure 3.**
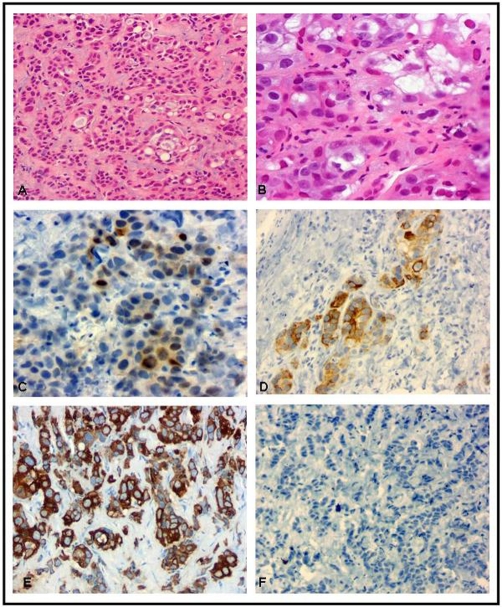
**A & B:** Pleural biopsy, H&E stain : There are nests, cords, and gland-like structures present **(A)**. Tumor cells are composed of epithelial-like cells with eosinophilic cytoplasm, marked nuclear pleomorphism, macronucleoli and occasional mitotic figures **(B)**. **C & D:** Immunohistochemistry: Tumor cells are focally positive for calretinin **(C)** and CK5/6 **(D)**. **E & F:** Immunohistochemistry: Tumor cells are strongly positive for CK7 **(E)** and negative for TTF-1 **(F)**.

H&E stain of the left posteroinferior pleura and right adrenal mass showed a malignant neoplasm consisting of papillary structures, branching tubules, and gland-like acinar and cystic spaces. These structures were lined by uniform cuboidal and flattened epithelial-like cells with large, round-to-oval, pleomorphic, vesicular nuclei, prominent macronucleoli, occasional mitotic figures and abundant eosinophilic cytoplasms with distinct cytoplasmic borders (see Figure 3A & B). Occasional mucin droplets were present. The surrounding matrix varied from myxoid to densely fibrous with hyalinized material with rare mitotic figures (1/10 hpf). Based on microscopic features, it was essential to distinguish malignant mesothelioma from pulmonary adenocarcinoma. This was achieved by immunohistochemical analysis.

Immunohistochemical (IHC) analysis of the left posteroinferior pleural and right adrenal masses showed similar immunoreactivity. Neoplastic cells were strongly positive for cytokeratin 7, focally positive for calretinin and cytokeratin 5/6, and negative for thyroid transcription factor-1 (TTF-1) (see Fig. 3 C-F). This profile was consistent with primary malignant mesothelioma of the left pleural mass and metastatic mesothelioma of the right adrenal mass. IHC analysis is typically used for diagnosing and differentiating mesotheliomas from other lung cancers. This requires the utilization of a panel of immunostains such as TTF-1, calretinin, CK5/CK6, MOC31, B72.3, and BG8. Anti-calretinin antibody is the most specific marker of epithelial mesothelioma, since it exhibits both cytoplasmic and nuclear distribution patterns. TTF-1 is found only in thyroid-derived tissue and lung carcinomas. CK5 and CK6 are strongly positive in epithelial mesotheliomas, while pulmonary adenocarcinomas are predominantly negative. Conversely, both lung carcinomas and mesotheliomas are strongly immunoreactive with CK7.

## Discussion

This 41-year old patient subsequently died due to complications from continued mesothelioma metastases and HIV/AIDS. The diagnosis of mesothelioma has traditionally posed a challenge even to experienced pathologist. This is because the epithelial-variant is histologically similar to adenocarcinoma. They can be effectively differentiated by IHC analysis. Undoubtedly, our patient had epithelial-variant mesothelioma, not adenocarcinoma, since IHC analysis confirmed the presence of calretinin, cytokeratin 5, 6 and 7, and the absence of TTF-1 normally present in adenocarcinoma.

### Etiology & Pathogenesis

The risk of developing mesothelioma may be increased by exposure to asbestos. However, a host of other factors may contribute to the occurrence of mesothelioma, including cigarette smoke, infection by immunomodulatory viruses SV-40 and HIV, and immunosenescence due to aging.

It is well documented that cigarette smoke increases the risk of airway cancers. Individuals with a history of smoking without asbestos exposure have an eleven-fold increase in relative risk of airway lung cancer. Conversely, when there is a history of asbestos exposure without cigarette smoking, there is only a five-fold relative risk of developing airway lung cancer. When combined, smoking and asbestos increase the relative risk of airway lung cancer by fifty-five folds[2]. However, evidence to support smoking as an increased risk specifically for mesothelioma formation is lacking. It has been shown that cigarette smoking increases the permeability of the lungs, making it easier for asbestos fibers to penetrate the lung parenchyma. Also, research has demonstrated that greater concentrations of asbestos fibers are retained in the lungs of smokers compared to non-smokers. Additionally, smoking dysregulates the mucociliary escalator, leading to diminished ability to clear foreign material (such as asbestos) from the airways[3]. Studies performed in rats showed that smoking enhances susceptibility to mesothelioma in animals with a history of asbestos exposure[4]. When considered together, these reports suggest the occurrence of enhanced mesothelioma-induction during dual-exposure to cigarette smoke and asbestos. However, more studies are needed.

It is possible that our patient was exposed to environmental asbestos in lieu of occupational asbestos, since this has been linked to the development of mesothelioma. However, the question of incubation time remains a point of conflict for this 41-year old patient, since progression to mesothelioma generally requires 30-50 years incubation. Interestingly, the polyoma virus SV-40 has been implicated as a participant in some cases of mesothelioma. It is postulated that since the virus inactivates anti-tumor genes such as retinoblastoma, it promotes immunosuppression that may lead to enhanced susceptibility to mesothelioma. Like SV-40 virus, HIV is also an oncovirus and therefore capable of inducing cancer. Since HIV suppresses the immune system by depleting immune cells, and produces neoplasms such as lymphomas in infected patients, we believe that HIV increases the susceptible to mesothelioma. Although not as prevalent in HIV-infected patients as Kaposi’s sarcoma and non-Hodgkin’s lymphoma, mesothelioma cases have been reported in this patient population without a history of asbestos exposure[5;6). Some credence to the role of immunomodulation has been provided by the detection of immunosuppressive mediators in pleural effusions that were located adjacent to mesothelioma cells. For example, it has been shown that mesothelioma cells can produce TNF-alpha, which promotes immunodysfunction that aids in protection of the malignant cells via NF-kB-mediated signaling[7].

Other immunosuppressed patients include transplant patients and elderly individuals. Transplant patients are immunosuppressed due to immunocytotoxic drugs implemented to prevent transplant organ rejection, while elderly patients undergo physiologic immunosenescence characterized by reduced cellular and humoral immune responses[8]. Not surprisingly, mesothelioma has been reported in transplant patients, in the absence of notable asbestos exposure[9], and mesotheliomas are classically reported in elderly patients.

In summary, these reports suggest that compared to the general population, mesothelioma may be more prevalent in SV-40 virus-infected patients, HIV/AIDS patients, organ transplant patients, and elderly patients. Even more interesting, these reports hint at the possible role of immunosuppression as a prerequisite for mesothelioma occurrence.

### Treatment and Prognosis

The prognosis for our patient could have been estimated by considering his age of less than 65-years, the epithelial-variant of mesothelioma identified, the patient’s serum lactate dehydrogenase value less than 500 IU/L, and the platelet counts less than 400 000/mm^3^. While these criteria suggested a favorable profile for mesothelioma that would be slowly progressive, the Butchart system suggested a grim prognosis since he had stage IV mesothelioma. His prognosis was guarded and his disease was predicted to progress to fatality in 6 to 18 months. Treatment with pemetrexed and cisplatin may reduce the rate of disease progression, while smoking cessation and early intervention with supplemental oxygen for hypoxemia may assist in symptom management. Influenza, pneumococcal and meningococcal vaccinations were recommended since he had increased risk for pneumonia. Finally, anti-retroviral therapy can provide immuno-cytoprotection that may reduce the rate of disease progression. The patient described in this report received all of the above treatments, however his disease was already too advanced and rapidly progressed to death.

## Conclusions

The major risk factor for the development of mesothelioma is exposure to asbestos, and disease typically occurs in elderly patients 30-50 years post-exposure. Disease manifestation involves progressive respiratory distress and chest pain that arise from pleural fibrosis/effusions. In severe cases, hypertrophic pulmonary osteo-arthropathy, Cor pulmonale and heart failure have occurred.

The development of mesothelioma in patients with HIV/AIDS, SV-40 infection, organ transplant, or advanced age suggests that chronic immunosuppression enhances susceptibility to mesothelioma. Since disease in many of these patients has been documented prior to the 5^th^ decade of life, it is plausible to suggest that immunosuppression either promotes or accelerates the development of mesothelioma, precluding the need for 30-50 years of incubation. This implies that immunocompetence in young asbestos workers may actually restrict the onset/progression of mesothelioma, therefore leading to a meager 8% prevalence in young asbestos workers.

Further research is needed in order to gain a better understanding of mesothelioma pathogenesis. In an effort to identify occupational mesothelioma earlier during disease progression, high-risk workers can be screened for abnormal serum osteopontin levels, since this is routinely elevated in mesothelioma. Although serum osteopontin may also be elevated in other forms of cancer, Pass *et al.* demonstrated that serum osteopontin has a 77.7% sensitivity and 85.5% specificity for mesothelioma[10]. Finally, we hope this report will stimulate clinicians to consider the possibility of mesothelioma in cases where lung cancer is coupled with immunosuppression.

## List of abbreviations

ADC: Adenocarcinoma
AIDS: Acquired immune deficiency syndrome
B72.3: immunohistochemical stain specific to pulmonary ADC not MM
BG8: Immunohistochemical stain specific to pulmonary ADC not MM
CK5/CK6: Cytokeratin 5/cytokeratin 6
HCO3: bicarbonate
HIV/AIDS: human immunodeficiency virus
H&E stain: Hematoxylin and eosin stain
IHC: immunohistochemical
IU/L: international units/liter
L: liter
mEq/L: milliequivalents/liter
MM: malignant mesothelioma
mmHg: millimeters of mercury
MOC31: immunohistochemical stain specific to MM not ADC
NF-kB: nuclear factor kappa-light-chain-enhancer of activated B cells
PaCO2: pressure of carbon dioxide
PaO2: pressure of oxygen
PET-CT: positron emission tomography- computed tomography
pH: potential of hydrogen
SaO2: saturation of oxygen in arterial blood
SV-40: simian virus-40
TNF-alpha: tumor necrosis factor-alpha
TTF-1: thyroid transcription factor-1
